# Comparative lipidomic and metabolomic profiling of mdx and severe mdx-apolipoprotein e-null mice

**DOI:** 10.1186/s13395-024-00368-w

**Published:** 2024-12-23

**Authors:** Ram B. Khattri, Abhinandan Batra, Zoe White, David Hammers, Terence E. Ryan, Elisabeth R. Barton, Pascal Bernatchez, Glenn A. Walter

**Affiliations:** 1 Department of Physiology and Aging, University of Florida, Gainesville, FL USA; 2 Department of Physical Therapy, University of Florida, Gainesville, FL USA; 3 Department of Pharmacology & Therapeutics, University of Florida, Gainesville, FL USA; 4 Department of Applied Physiology and Kinesiology, University of Florida, Gainesville, FL USA; 5 Department of Anesthesiology, Pharmacology & Therapeutics, University of British Columbia, and Centre for Heart + Lung Innovation, St Paul’s Hospital, Vancouver, BC Canada; 6 Center of Exercise Science, University of Florida, Gainesville, FL USA; 7 Department of Physical Therapy, University of Louisiana, Monroe, LA USA; 8 Department of Physiology and Aging, University of Florida, PO BOX 100274, Gainesville, FL 32610 USA

**Keywords:** Duchenne, Fibrofatty infiltration, Metabolism, Muscle, Liver, NMR, HR-MAS

## Abstract

Despite its notoriously mild phenotype, the dystrophin-deficient *mdx* mouse is the most common model of Duchenne muscular dystrophy (DMD). By mimicking a human DMD-associated metabolic comorbidity, hyperlipidemia, in *mdx* mice by inactivating the apolipoprotein E gene (*mdx-ApoE*) we previously reported severe myofiber damage exacerbation via histology with large fibro-fatty infiltrates and phenotype humanization with ambulation dysfunction when fed a cholesterol- and triglyceride-rich Western diet (*mdx-ApoE*^W^). Herein, we performed comparative lipidomic and metabolomic analyses of muscle, liver and serum samples from *mdx* and *mdx-ApoE*^W^ mice using solution and high-resolution-magic angle spinning (HR-MAS) ^1^H-NMR spectroscopy. Compared to *mdx* and regular chow-fed *mdx-ApoE* mice, we observed an order of magnitude increase in lipid deposition in gastrocnemius muscle of *mdx-ApoE*^W^ mice including 11-fold elevations in -CH_3_ and -CH_2_ lipids, along with pronounced elevations in serum cholesterol, fatty acid, triglyceride and phospholipids. Hepatic lipids were also elevated but did not correlate with the extent of muscle lipid infiltration or differences in serum lipids. This study provides the first lipometabolomic signature of severe mdx lesions exacerbated by high circulating lipids and lends credence to claims that the liver, the main regulator of whole-body lipoprotein metabolism, may play only a minor role in this process.

**Supplementary Information:**

The online version contains supplementary material available at 10.1186/s13395-024-00368-w.

## Introduction

Duchene muscular dystrophy (DMD) is the most prevalent inherited muscle disorder [1]. The development of therapeutic interventions for DMD has been hampered by the major differences in disease presentation between DMD patients and animal models [2]. Over sixty different dystrophin-deficient animals have been developed to study the pathogenesis of DMD although with mixed relevance to the human condition [3]. A commonly used one is the *mdx* mouse with a mutation in the X-linked *DMD* gene, which encodes for dystrophin [4]. However, the robust regenerative response typically found in mouse muscles results in a notoriously mild phenotype with only minor skeletal muscle fibrofatty filtration at a much later stage [5] than what is observed in DMD patients [6–8].

A recent advance in DMD modeling is the *mdx*-4CV mouse lacking apolipoprotein E (*mdx-ApoE*) with elevated levels of blood lipoprotein-associated cholesterol and triglycerides when on a Western diet (*mdx-ApoE*^W^), a key metabolic comorbidity of DMD, hyperlipidemia. Indeed, we reported that DMD patients are afflicted by a new form of hyperlipidemia with high circulating TG and nonHDL-associated cholesterol that is not secondary to the muscle wasting process or steroids use [9]. Similar to the human DMD, *mdx-ApoE*^W^ mice show large fibrotic and fatty infiltration in triceps and gastrocnemius muscles, severe muscle atrophy and ambulation dysfunction [6], which supports the concept of treating DMD patients with cholesterol-lowering medications. MRI imaging has further documented fibrofatty infiltration in appendicular muscles of *mdx-ApoE*^W^ [10], through increased inflammatory and apoptosis signaling. Age-dependent changes in metabolomic and lipidomic profiling were reported in mild *mdx* and control tissue and serum samples [1]. Similarly, longitudinal elevations in serum and plasma glycerolipids and glycerophospholipids families have been reported in *mdx* mice using liquid chromatography-mass spectrometer (LC-MS), although the relevance to the more severe disease etiopathology of human DMD is unclear [11].

Herein, we performed comparative metabolomic and lipidomic analyses in tissues and serum samples from *mdx* and *mdx-ApoE* mice fed regular chow (R) or Western (W) diets using high-throughput analytical techniques. We performed high-resolution magic angle spinning (HR-MAS) ^1^H spectroscopy, a variant of nuclear magnetic resonance (NMR) [12–14] that allows for the non-destructive quantification and characterization of small metabolites and water-insoluble metabolites in semi-solid samples [15, 16], making it suitable for metabolomics profiling [17–20] of muscle and the liver; and high resolution solution ^1^H NMR of serum. We provide the first molecular signature of how elevated circulating lipids exacerbate dystrophin-deficient muscle wasting and suggest that the liver, the main regulator of whole-body cholesterol metabolism, may play a secondary role in this process.

## Results

### NMR

**Fig. 1:**
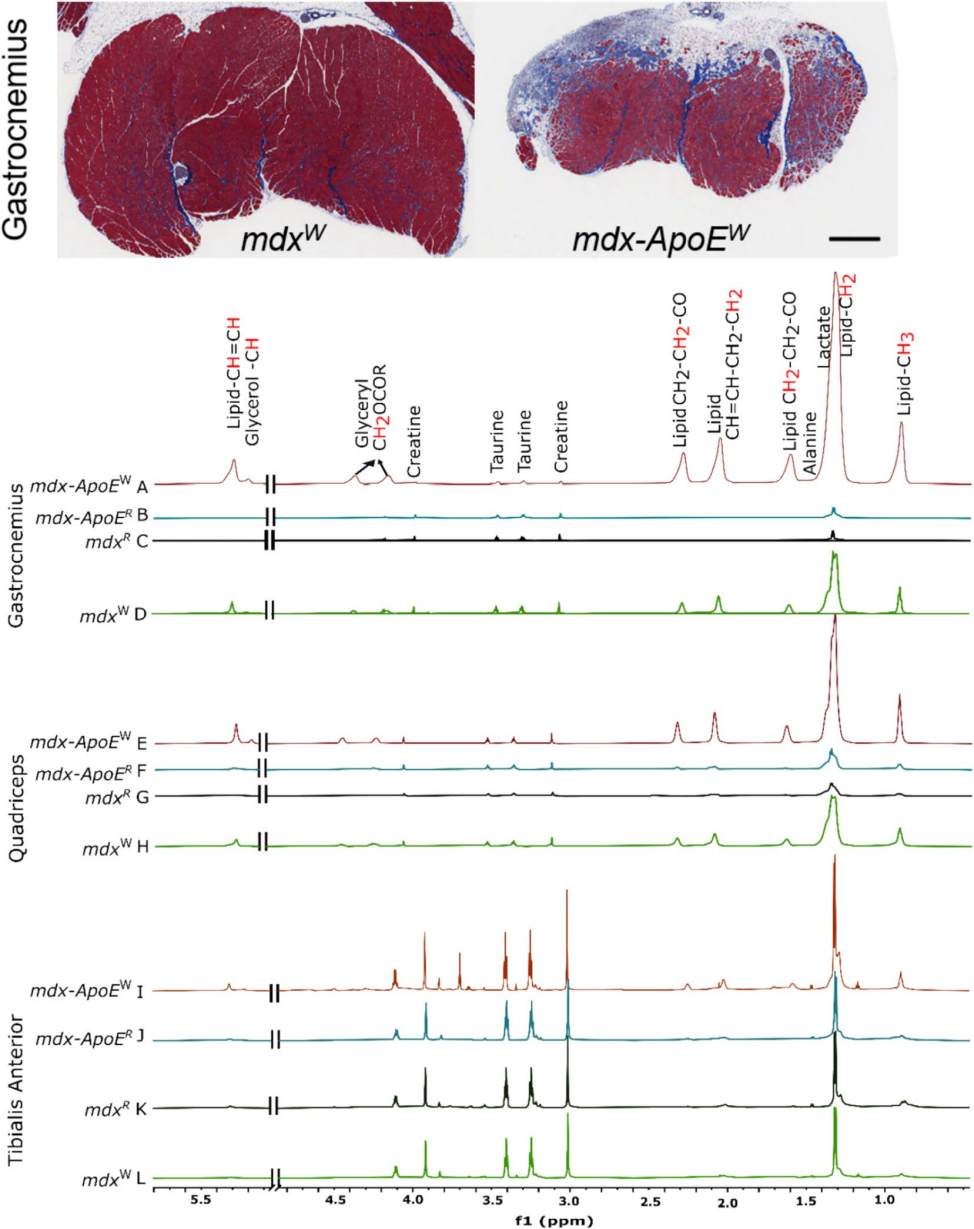
Representative Mason’s trichrome image of severely affected gastrocnemius muscle from Western diet (W)-fed mdx and mdx-ApoE KO mice, scale bar 1 mm. HR-MAS spectra for gastrocnemius, quadriceps and tibialis anterior muscle samples (aliphatic region) showing lipids and small metabolites resonance for four different groups: (1) *mdx-ApoE*^W^ (brown; **A**, **E**, & **I**), (2) *mdx-ApoE*^R^ (blue; **B**, **F**, & **J**), (3) *mdx*^R^ (black; **C**, **G**, & **K**), and (4) *mdx*^W^ (green; **D**, **H**, & **L**)

**Fig. 2:**
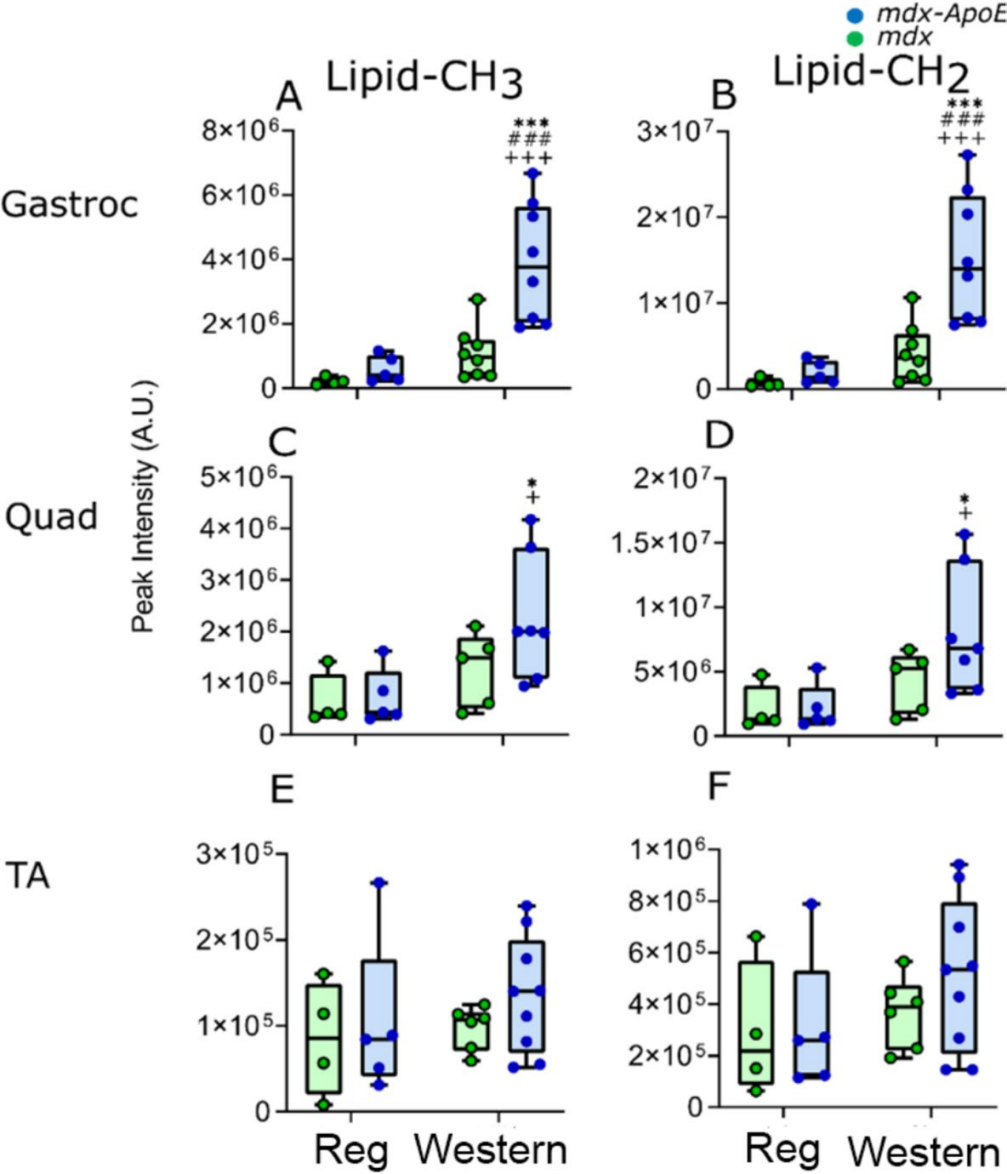
Box and Whisker plots showing relative abundance of lipid CH_3_ and lipid CH_2_ in gastrocnemius (Gastroc), quadriceps(Quad), and tibialis anterior muscles (TA) via ^1^H HR-MAS spectra. Significance was determined by two way ANOVA with “*p*” = 0.05 − 0.01, “*p*” ≤ 0.05 is denoted with “*”/”^#^”/”^+^”, “*p*” between 0.01 − 0.001 is denoted with “**”/”^##^”/”^++^”, and “*p*” ≤ 0.001 is denoted with “***”/”^###^”/”^+++^”. “Asterisks” means significantly different within the same strain, “hash tag” means significantly different than the other strain within the regular (Reg) diet group, and “plus” means significantly different than the other high-fat Western diet strain. Numbers were *mdx-ApoE*^R^ (*n* = 5), *mdx-ApoE*^W^ (*n* = 7–9), *mdx*^R^ (*n* = 4), and *mdx*^W^ (*n* = 5–8)

HR-MAS of tissues reveals higher lipid levels in Western diet-fed groups. ^1^H HR-MAS spectra were acquired from gastrocnemius (gastroc), soleus (Sol), extensor digitorum longus (EDL), tibialis anterior (TA), quadriceps (quad), and liver tissues of chow- or Western diet-fed mdx and mdx-ApoE mice. Figure 1 shows the representative aliphatic region of HR-MAS spectra for the gastrocnemius (severely affected; Fig. 1A-D) with Mason’s trichrome histology, quadriceps (mildly affected; Fig. 1E-H), and TA (mostly unaffected; Fig. 1I-L) muscle samples showing lipids and small metabolites resonance (with annotations) of four different groups. Similar spectra were obtained for Sol (Supplementary Figure S1 A-D), EDL (Supplementary Figure S1 E-H), and liver tissues (Supplementary Figure S1 I-L). Quantification revealed that lipid-CH_3_ (peak integration area between 0.83 and 0.96 ppm Figs. 1 and 2) and lipid-CH_2_ (peak integration area between 1.19 and 1.40 ppm, which represents lipoproteins) levels were significantly higher in mdx-ApoE^W^ gastroc, a highly exacerbated muscle by circulating lipids, showing 11-fold increases in both lipid-CH_3_ and -CH_2_ signals of compared with mdx-ApoE^R^ (*p* < 0.001) and other groups (Fig. 2A & B, respectively), a sign of lipid deposition, whereas moderately exacerbated quad samples showed more moderate increases (Fig. 2C & D; *p* < 0.05) for both lipid types. In contrast the non-lipid-exacerbated TA (Fig. 2E & F, respectively), EDL and Sol muscles (Supplementary Figure S2 A-D) showed minor changes in lipid accumulations. However, mdx-ApoE^W^ liver samples unexpectedly showed significantly lower lipid-CH_3_ as well as lipid-CH_2_ content than samples from mdx mice when fed a Western diet (with p-value in between 0.05 and 0.0001; Supplementary Figure S2 E & F), suggesting that loss of ApoE in mdx mice on a Western diet has opposite effects on lipid accumulation in the liver and severely affected muscles.

**Fig. 3:**
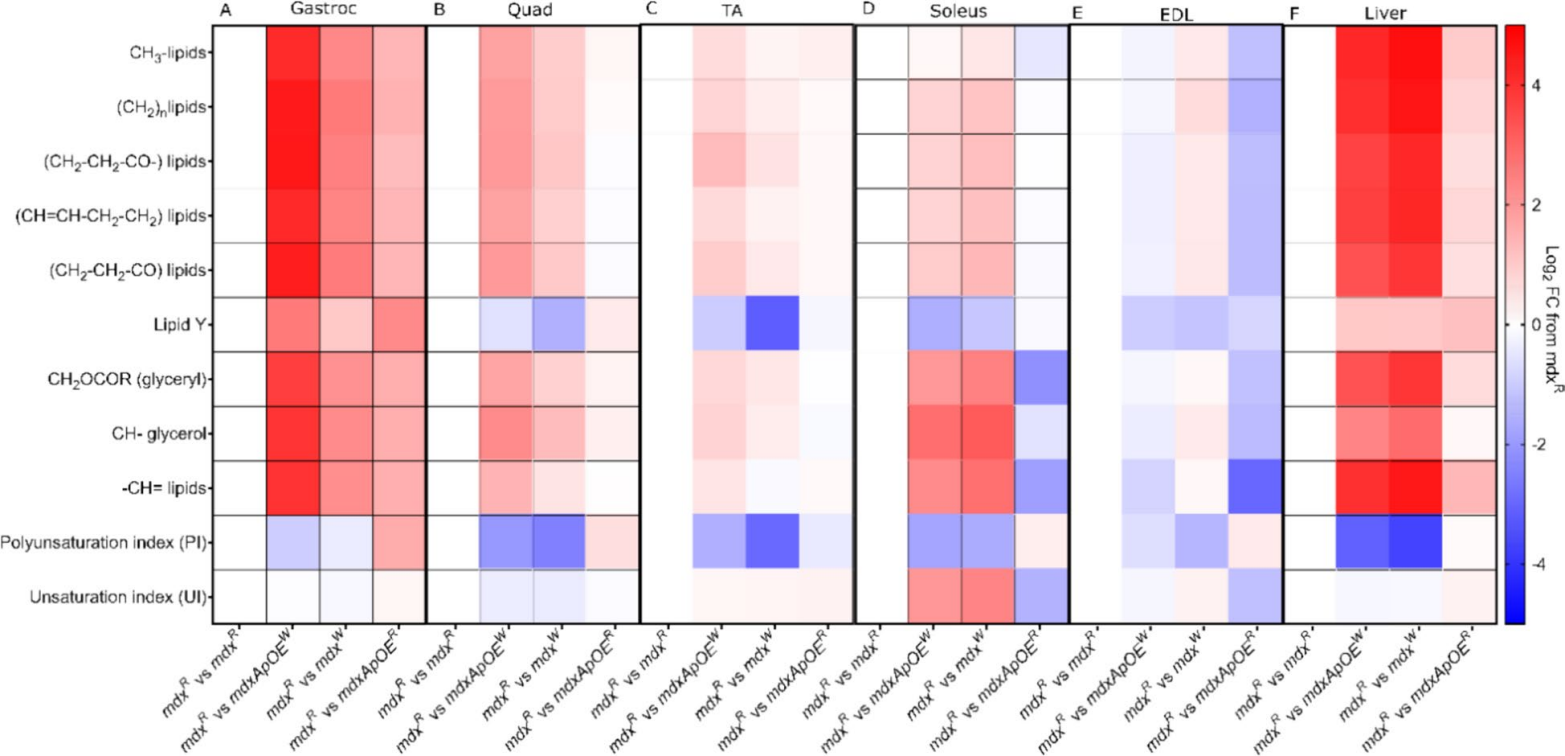
Heatmap representing the log_2_-transformed fold changes with respect to *mdx*^*R*^ group for few lipid classes in six tissue samples obtained via ^1^H HR-MAS spectra. **A** Gastroc, **B** Quad, **C** TA, **D** Soleus, **E** EDL, and **F** Liver. The number of samples per group were as follows: *mdx-ApoE*^R^ (*n* = 5), *mdx-ApoE*^W^(*n* = 7–9), *mdx*^R^(*n* = 4), and *mdx*^W^(*n* = 5–8). Gastroc: gastrocnemius, quad: quadriceps, TA: tibialis anterior, EDL: extensor digitorum longus, and FC: fold-change

Heat map representing the log_2_-transformed fold changes of lipid classes compared with mice on regular diet (*mdx*^R^) mice revealed that the gastroc (Fig. 3A) and liver (Fig. 3F) display larger changes in lipids abundance than other tissues, the latter mostly influenced by the Western diet rather than loss of ApoE, whereas quads and TA (Fig. 3B-C) showed minor changes although the EDL showed the opposite trend to other muscles. The lipid polysaturation index (PI) followed similar trends along the all six tissue samples, decreasing in high-fat diet groups. The lipid unsaturation index (UI) was also decreased in high-fat diet groups for the tissues except soleus and EDL muscles (Supplementary Figure S4). Finally, metabolite analyses (Supplementary Figure S3) of the taurine to total creatine ratio (muscle regeneration), glucose, lactate (a glycolytic metabolite) and alanine (a non-essential amino acid that participates in sugar and acid metabolism) revealed only minor differences between groups in the EDL.

### Loss of ApoE has a greater effect on mdx extracted serum lipids than a western diet by solution state NMR

**Fig. 4:**
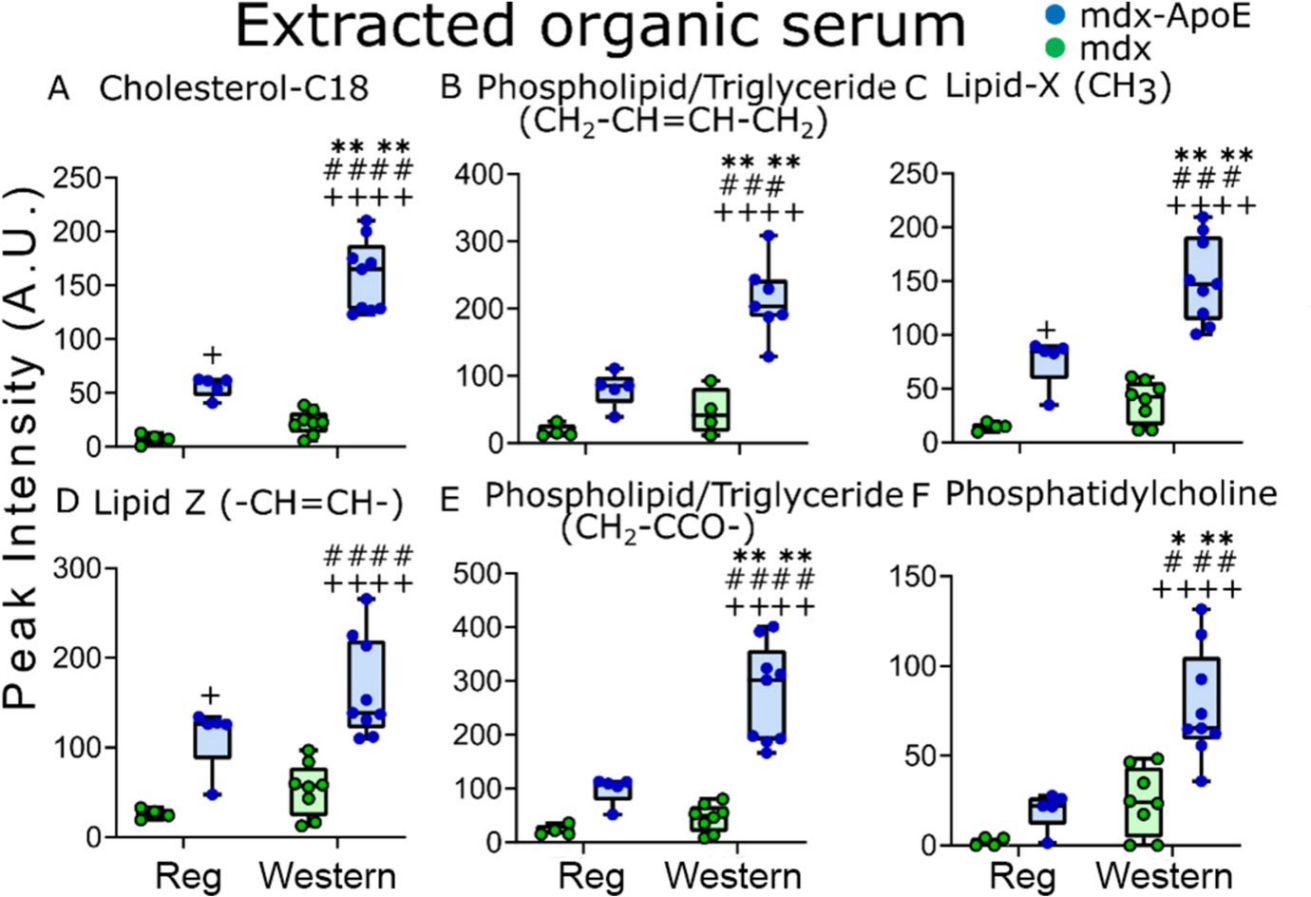
Representative Box and Whisker plots (with error bars) for lipid components obtained from ^1^H NMR spectra of organic phase extract of serum for four diet-dependent groups of *mdx* mice: **A** Cholesterol/cholesterol ester (CH_3_, C18), **B** Phospholipid/Triglyceride (-CH_2_-CH=CH-CH_2_), **C** Fatty acids (terminal CH_3_), **D** Fatty acids (-CH=CH-) **E** Phospholipid/Triglyceride (CH_2_-CCO-), and **F** Phosphatidylcholine (N^+^(CH_3_)_3_). Significance was determined by two way ANOVA with “*p*” = 0.05-0.01, “*p*” ≤ 0.05 is denoted with “*”/”^#^”/”^+^”, “p”≤ 0.001 is denoted with “***”/”^###^”/”^+++^”, and “p” ≤ 0.0001 is denoted with “****”/”^####^”/”^++++^”. “Asterisk/s” means significantly different within the same strain, “hash tag” means significantly different than the other strain within the regular diet group, and “plus” means significantly different than the other high-fat diet strain. The number of samples per group were as follows: *-ApoE*^R^ (*n*=5), *mdx-ApoE*^W^(*n*=9), *mdx*^R^(*n*=4), and *mdx*^W^(*n*=8)

Despite identifing differences in certain lipids (Supplementary Figure S5) we found major lipid differences were obscured by the presence of fatty acid binding proteins in the serum (Supplementary Figure S6). Using a modified Folch extractions to remove lipoproteins, clear differences were found in cholesterol (Chol-C18), triglycerides (TG), saturated and unsaturated fatty acids, phosphatidylcholines (Pt) and phospholipids (PL), supporting similar lipid profiles between severely-affected *mdx-ApoE*^W^ gastroc muscles and serum (Fig. 4A-F).

**Fig. 5:**
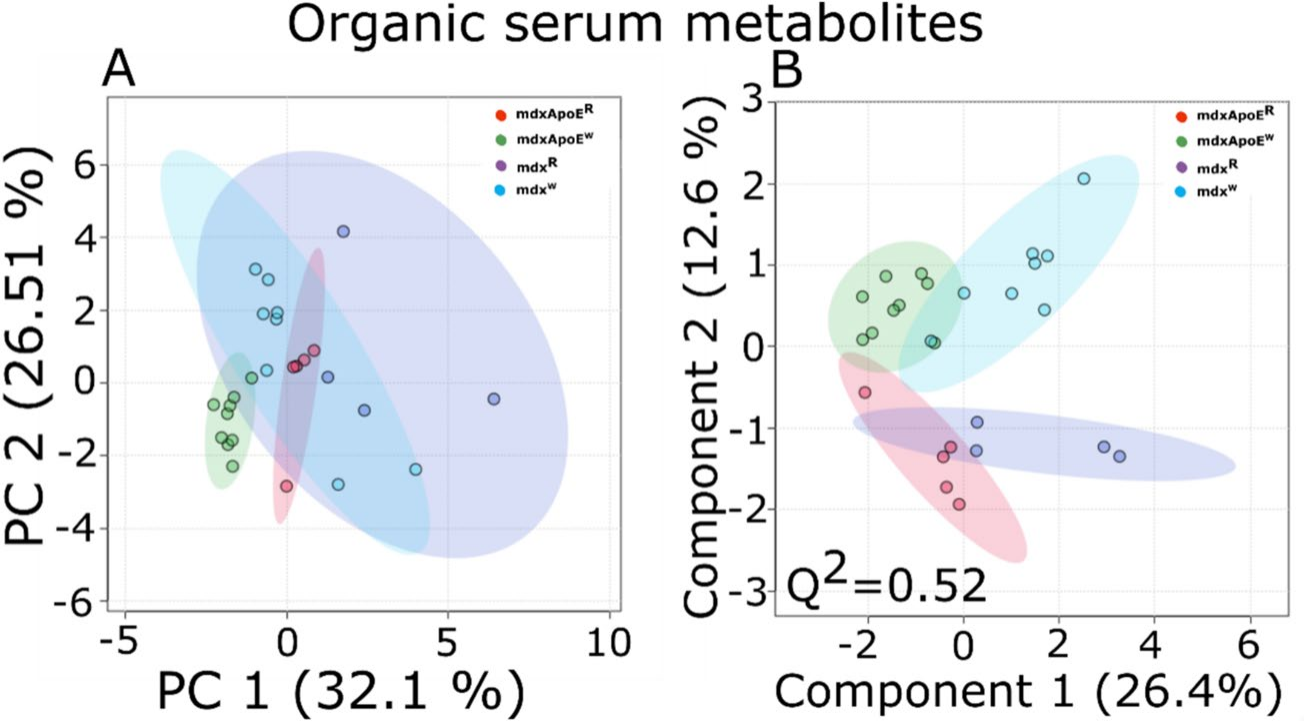
^1^H NMR metabolomics profiles for four diet-dependent groups of *mdx* mice for organic phase sera samples obtained from: **A** principal component analysis **B** Partial least square discriminant analysis. *mdx-ApoE*^R^ (red), *mdx-ApoE*^W^(green), *mdx*^R^ (blue), and *mdx*^W^ (cyan). Number of samples per group were as follows: *mdx-ApoE*^R^ (*n* = 5), *mdx-ApoE*^W^(*n* = 9), *mdx*^R^(*n* = 4), and *mdx*^W^(*n* = 8). PC: principal component

Principal component (PCA) and partial least square discriminant (PLS-DA) analyses on ^1^H NOESY spectra revealed separate clustering among *mdx-ApoE*^R^, *mdx-ApoE*^W^, and *mdx*^R^ groups. However, some overlapping can be observed for *mdx-ApoE*^R^, *mdx*^R^, and *mdx*^W^ (Fig. 5A) groups. Components 1 and 2 comprise 58.61% of the total variance among these four groups. The supervised method PLS-DA supports the PCA finding and a more pronounced clustering among the four groups can be observed, with 39% of the total variance among the groups are explained by components 1 and 2 (Fig. 5B). Permutation test also showed a Q^2^ > 0.5. Table 1 comprises the list of chemical shifts compelling separation among the groups obtained from VIP-plot of PLSDA analysis for the lipid phase serum samples.

**Table 1:** VIP score plot for extracted lipid phase serum samples obtained from VIP-plot of PLSDA analysis

Spectra range (ppm)	Lipid class	Associated protons	Peak Pattern	VIP Scores
1.54–1.64	Phospholipid/Triglyceride	(C**H**_**2**_-CH_2_-COO)Ph, TG	m	2.25
1.98–2.01	Phospholipid/Triglyceride	(C**H**_**2**_-CH = CH-CH_2_)	m	1.75
3.51–3.55	Unknown	Unknown	m	1.5
5.29–5.42	Fatty acids	(-C**H** = C**H**-) FAs	dd	1.45
4.55–4.66	Unknown	Unknown	m	1.4
0.87–0.92	Fatty acids/cholesterol	C**H**_**3**_	m	1.32
1.04–1.19	Cholesterol	C**H**_**3**_	m	1.28
2.74–2.78	Phospholipids, Triglyceride	(CH-CH-C**H**_**2**_-CH-CH)	m	1.22
3.48–3.56	Phosphatidylcholine	(-CH_2_-N)	s	1.01
1.20–1.39	Fatty acids /Triglyceride /Phospholipids	(-C**H**_**2**_-C**H**_**2**_-C**H**_**2**_-)	Complicated s	0.7
0.84–0.87	Cholesterol	Chol-C**H**_**3**_ (C26, C27)	dd	0.68
4.25–4.34	Triglyceride	(-C**H**_**2**_**-)**	dd	0.5
3.67–3.83	Phosphatidylcholine	(3CH_2_-)	s	0.21
0.87–0.89	Cholesterol	Chol-C**H**_**3**_ (C19, C21)	d	0.20

Abbreviations used to describe the resonance splitting patterns

resonance: *m* multiplet, *dd* doublet of doublets, *s* singlet

**Fig. 6:**
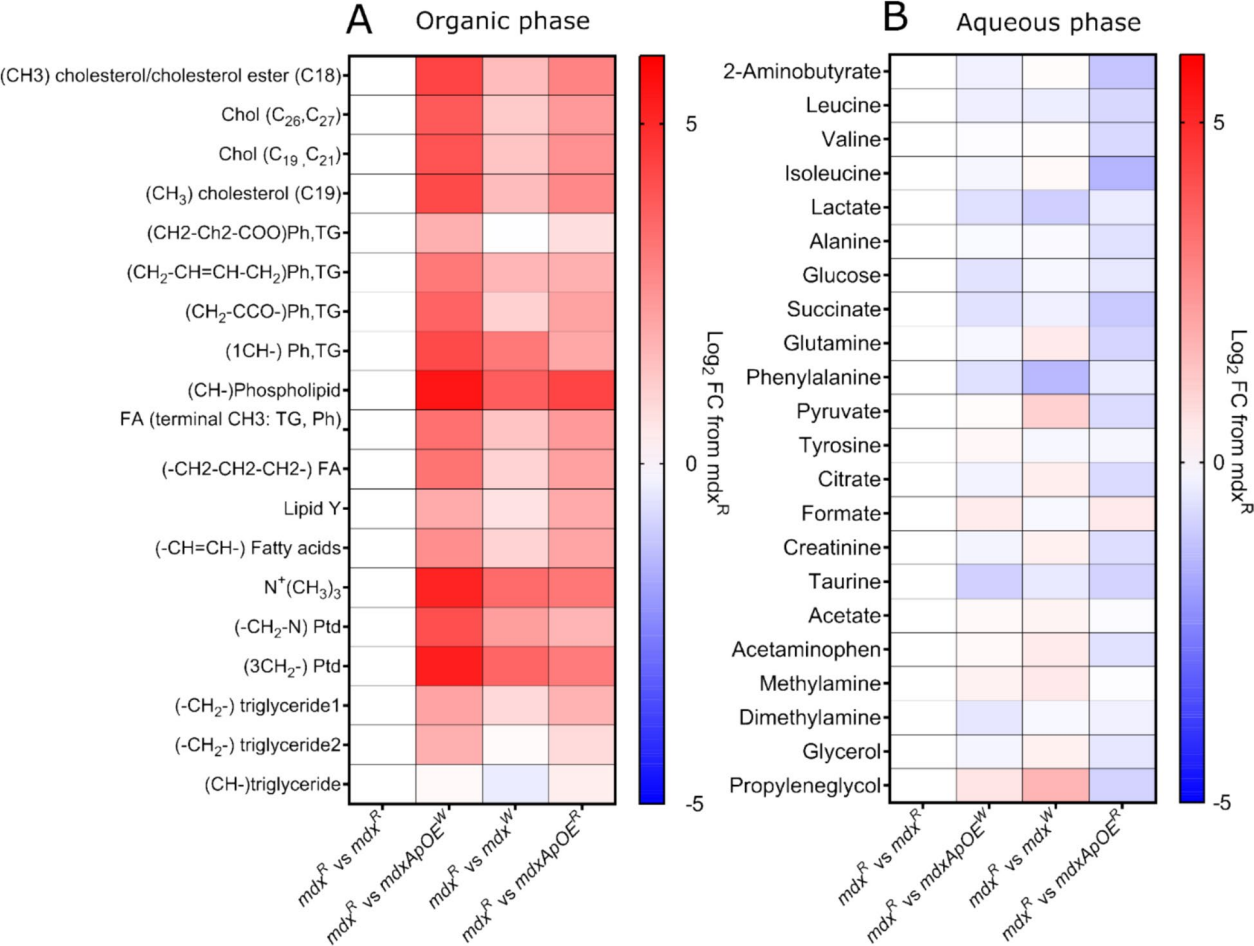
Heat maps representing the log_2_-transformed fold changes compared with the *mdx*^R^ group for organic and aqueous phase serum samples obtained via 1D ^1^H NOESY spectra. **A** organic phase, **B** aqueous phase. FC: fold-change

Clustering between the groups were observed for aqueous phase serum samples with both PCA (components 1 and 2 comprising 61.3% of the total variance) and PLS-DA (with 52.1% of the total variance in components 1 and 2) analysis (Supplementary Figure S7 A & B, respectively). The Q^2^ value for PLS-DA was below 0.4. Only lactate showed VIP scores value greater than 1.6 in VIP scores plot obtained from PLS-DA analysis (Supplementary Table ST1) whereas little changes were observed for leucine, isoleucine, valine, glucose, lactate and alanine (Supplementary Figure S8 A-F). Heat maps comparing log_2_-transformed fold changes in organic and aqueous phase samples compared with *mdx*^R^ mice revealed larger magnitude changes in lipid compound’s abundance in the more severe *mdx-ApoE*^W^ group compared to other three groups (Fig. 6A). Water-soluble metabolites showed subtle changes among the four groups (Fig. 6B).

## Discussion

DMD is a complex neuromuscular disease with a growing number of co-morbidities. While these may be caused by steroids, hyperlipidemia has been documented following the loss of a single dystrophin allele, which does not cause muscle wasting in carrier individuals [9]. As mice are notorious for their nonHDL-poor circulating lipid profile, we have previously demonstrated humanization of the *mdx* mouse phenotype via ApoE gene inactivation and Western diet supplementation, as well as rescue with cholesterol absorption blocker ezetimibe, directly implicating nonHDL as a critical disease-exacerbating factor [6, 21]. With the current report rely on advanced analytical approaches to characterize the differences in lipometabolic signatures between severely- (gastroc), moderately (quads) and mildly-exacerbated muscles (soleus, TA, EDL) in function of circulating lipid profiles, it is likely that dystrophin-deficient muscles have lost the ability to regulate their intramuscular lipid content, particularly cholesterol as recently suggested by others [22], leading to large fibrofatty infiltrates [23–27]. While this could be tested in patients with cholesterol-targeting medications, whether this is a primary driver of myofiber loss or secondary consequence is poorly understood [6]. Indeed the rate that the muscle is replaced by fat has become a viable MRI biomarker to detect early disease progression in DMD and the serum from DMD boys can be characterized by dyslipidemia at an early age [9]. In this study we aimed to determine if the *mdx-ApoE* mouse on a Western diet recapitulated some of the lipid changes observed in DMD while providing the opportunity to compare lipid profiles in multiple muscles, blood, and the liver. We found there was not only a difference in total lipid content between *mdx* and *mdx-ApoE* mice but these differences were excaberated by a western diet. In muscular dystrophy, it has been suggested that altered lipid metabolism affects the balance of saturated and unsaturated fatty acids in the muscle [1, 28] and blood [1, 29]. In both DMD [28] and in *mdx* mice [1] reduced levels of linoleic acid (an essential polyunsaturated fatty acid) are observed in affected muscle tissue and this imbalance in fatty acid saturation may contribute to muscle cell injury and dysfunction [28]. These fatty acid differences appear to be pushed futher out of balance in some muscles. This may account for the increased phospholipid/triglycerides that are observed in the blood and a major class of lipids that distinquished differences among all four animal groups.

Unsaturation and poly-unsaturation indices are the parameters that were utilized to characterize lipid composition in this study. The degree of unsaturation is correlated to the vinylic protons at 5.4 ppm, whereas polyunsaturation index correlated with diallylic protons at 2.75 ppm. From a biological perspective, the results obtained for PI and UI in this study indicate that lipid levels are qualitatively different in all four *mdx* and *mdx-ApoE* groups with different diet regimens. Altogether, decrease in the PI and UI values were observed for Western high fat diet groups (in both *mdx* and *mdx-ApoE*) compared to normal diet groups for most of the tissue samples under investigation. Interestingly, even in muscles in which there was not a difference in total lipid deposition, such as the solues, there were striking differences in the lipid PI.

One of the lipids of interest we identified in exacerbated *mdx-ApoE*^W^ muscles is cholesterol, one of the critical components of muscle cell membranes. Cholesterol is highly enriched in the T-tubule of myofibers and appears to be the key culprit behind disease exacerbation caused by ApoE inactivation in both DMD and Dysferlinopathies as cholesterol absorption blocker ezetimibe drastically reduces disease severity [21]. While it is reasonable to speculate that muscle cholesterol accumulation is of lipoprotein origin, upregulated HMGCoAR expression has been observed in *mdx* and dysferlin-null muscle tissues along with free cholesterol [22, 30]. How extramyofiber cholesterol accumulation differs from intramyofiber cholesterol accumulation warrants further investigation.

While serum metabolite levels showed overall decreases in severely-affected *mdx-ApoE*^*W*^, a likely consequence of decreased muscle mass, an unexpected observation was that changes in muscle lipids did not fully correlate with hepatic lipids. Despite being the main regulator of whole-body cholesterol and lipoprotein homeostasis, the hepatic contribution to DMD-associated dyslipidemia and exacerbation of muscle wasting in *mdx-ApoE* mice is unknown. Others have shown metabolic abnormalities in *mdx* liver samples, although we did not observe free cholesterol accumulation or HMGCoAR up-regulation in dysferlin-deficient liver samples, in stark contrast to muscle tissues [31]. Herein, similarly to muscle samples, *mdx-ApoE*^W^ liver lipids signatures were not as exacerbated as *mdx-ApoE*^R^ signatures, suggesting that the liver may only play a secondary role in regulating DMD lipoprotein abnormalities, and instead that muscle tissues may directly interfere with circulating lipids homeostasis. Future experiments aimed at imaging ^13^C-glucose and ^2^H-fatty acid metabolism in multiple organs through isotopic flux measurements may shed light on how DMD modulates the muscle-lipoprotein-liver interplay.

In conclusion, we observed major changes in the lipid and metabolite composition of serum and tissue samples of *mdx* and *mdx-ApoE* mice on normal and Western high fat diet regime. Lipid content and saturation was altered significantly in serum and in most of the tissue samples under investigation and therefore, could be considered a potential biomarker to monitor disease progression. The *mdx-ApoE*^W^ group were found to have significantly higher fat infiltration in tissue samples and serum, mimicking human condition more closely, and thus, can be considered more appropriate preclinical model for DMD disease.

## Materials and methods

### Animals

The study was approved by the University of Florida (Gainesville, FL) and University of British Columbia Institutional Animal Care and Use Committees. A total of nine *mdx* mice and seventeen *mdx-ApoE* KO mice were used for the study. The experimental mice were generated as explained previously by Milad et al. (2017) [6]. All mice were 32 weeks old at the start of the study and were fed either a Western diet (Harlan, TD88137; composition by weight: 0.2% total cholesterol, 21% total fat, and 34% sucrose) or control LabDiet 5001 or Envigo 7917 diet (Madison, WI) starting at 8 weeks of age till end of the study. Mice were housed in a regulated Association for Assessment and Accreditation of Laboratory Animal Care accredited facility (12-h light/dark, 22 °C, 42% humidity) and provided food ad libitum. Both male and female litters were used for the study with mice divided into four groups as follows: *mdx* mice on regular diet (*mdx*^R^ or *mdx*; *n* = 4), *mdx-ApoE* mice on regular diet (*mdx-ApoE*^R^;*n* = 5), *mdx* on Western diet (*mdx*^W^ ;*n* = 8) and *mdx-ApoE* on western diet (*mdx-ApoE*^W^; *n* = 9).

### Chemicals

All chemicals in this study, unless otherwise specified, were used as obtained from their respective vendors. D_6_−4,4-dimethyl-4-silapentane-1-sulfonic acid (DSS-D_6_, 98%) and deuterium oxide (D_2_O, 99.9%) were purchased from Cambridge Isotope Laboratories (Andover, MA, USA). Deuterated chloroform (99.8%) was manufactured and sold by Acros Organics (NJ, USA). Pyrazine, sodium azide (NaN_3_), sodium monobasic and dibasic phosphates, isonicotinic acid, and ethylene diamine tetra-acetic acid (EDTA) were obtained from Sigma Aldrich (St Louis, MO, USA). Methanol (99.9%) and chloroform (99.9%) were procured from Fisher Scientific, (NJ, USA).

### Solution NMR of serum

Serum NMR were acquired either with a Bruker (Bruker BioSpin Corporation, Billerica, MA) Avance Neo 600 MHz console and a Magnex 600/54-mm magnet equipped with an auto tune and match 1.7 mm TCI CryoProbe, or a Bruker (Bruker BioSpin Corporation, Billerica, MA) Avance 800 MHz console equipped with 54-mm magnet with an auto tune and match 5 mm TCI CryoProbe, at 25 ^o^C.

#### Whole serum extraction and preparation

Blood was collected from anesthetized mice (with ketazine/xylazine), via cardiac puncture. Tissues were processed for histology as previously published [6] or snap-frozen. To separate serum from blood, the collected blood was allowed to sit for 30 min at room temperature, after which it was centrifuged 15 min at 2,500 g at 25 ^o^C. The separated serum sample was then collected into a new Eppendorf tube, flash-frozen in liquid nitrogen, and stored in -80 ^o^C until the day of the extraction. NMR samples were prepared by mixing 39.25 µL of ice thawed serum with 3.75 µL of 1 M phosphate buffer (pH 7.2), 5 µL of Chenomx standard (DSS-D_6_ and 0.2% sodium azide (NaN_3_); Chenomx, Inc., Alberta, Canada]. 2 µL of 50 mM isonicotinic acid was added as the internal standards prepared in 99.9% D_2_O. 35 µL of the above sample mixture was loaded in a 1.7 mm NMR tube (CortecNet Corp, Brooklyn, NY, USA). The sample was prepared five minutes prior to the NMR experiment to maximize sample stability.

#### Extracted serum NMR

The lipid soluble and water-soluble metabolites from the serum sample were separated using modified Folch extraction. The modified Folch extraction was performed following the procedures mentioned previously by few literatures [15, 32, 33]. In short, extraction was performed using 39.25 µL of thawed serum sample in a glass vial. Dried aqueous and organic phases samples were obtained with this extraction method. The organic phase sample was re-suspended in 80 µL of CDCl_3_ consisting of 10 mM of pyrazine (as internal standard).

#### Solution-state NMR spectroscopy

Two sets of experiments were performed on the whole serum sample: 1D nuclear Overhauser effect spectroscopy (NOESY) [19, 34–37], and 1D periodic refocusing of J-evolution by coherence transfer (PROJECT) spectra [38]. The 1D ^1^H NOESY spectra were utilized for the quantification of the small metabolites.1D ^1^H spectra were acquired for extracted samples using 1D NOESY and PROJECT pulse sequence. 1D NOESY spectra were collected using first slice of NOESY pulse sequence (noesypr1d) applying a 90-degree pulse width (pw) with 1 s inter-pulse delay (d1) for 4 s acquisition time (at). The mixing time used was 100 ms with 128 scans (nt) in 7142.9 Hz spectral width (sw) [19, 34, 39]. PROJECT NMR spectra were collected with a 90-degree pw, sw = 7142.9 Hz, d1 = 3 s, 4.5 s “at”, loop size (L4) of 32 and 128 scans. Pre-saturation power was applied during “d1” and mixing time delays to suppress water signals for 1D NOESY and during “d1” for 1D PROJECT experiments.

### HR-MAS NMR on ex vivo muscle tissue samples

High-resolution ^1^H magic angle spinning (HR-MAS) was done to acquire high-resolution NMR data on 15 mg of intact gastroc, soleus, EDL, quad, TA, and liver tissues. Bruker 4 mm HRMAS probe was used on an 800 MHz and/or 600 MHz Bruker spectrometer (Topspin 3.5pl7 software) to acquire high-resolution NMR data on intact tissues. The HRMAS samples were prepared as described previously [15, 40]. A 3.2 mm inside diameter plastic insert was used to reduce the sample volume to approximately 50 µL, and confine the tissue to the center of the rotor to improve the shimming and reduce the centripetal forces that lead to tissue breakdown. The samples were spun at 5 kHz (at 54.7^o^ magic angle) maintaining its temperature of 4 ^o^C. Both 1D NOESY as well as PROJECT NMR spectra were acquired with “d1” of 0.8 s, 2.73 s “at”, 256 “nt”, 6009.6 Hz “sw” with 90-degree “pw” in common. The mixing time used in 1D ^1^H NOESY NMR was 100 ms, whereas loop number (L4) used in PROJECT NMR was 32. Weight wet of the tissues were utilized for normalization. Pre-saturation power was applied during “d1” and mixing time delays to suppress water signals for 1D NOESY and during “d1” for 1D PROJECT experiments.

### Data processing

All 1D and 2D NMR spectra were processed using MestReNova 14.0.1–23559 (Mestrelab Research, S.L., Santiago de Compostela, Spain). Spectra were apodized, zero filled to 65,536 points, Fourier-transformed, exponential line broadened to 0.3 Hz, phased, Spline and/or Whittaker Smoother baseline-corrected and calibrated. Proton spectra for whole serum and HR-MAS muscles’ spectra were calibrated to the alanine doublet at 1.46 ppm. Lipid phase 1D ^1^H spectra were referenced to chloroform at 7.26 ppm and normalized to the pyrazine (internal standard) peak at 8.59 ppm. Either integrated areas or mixed Gaussian/Lorentzian shape fitted peak multiplets areas were extracted for some selected metabolites using MestReNova and wet weight correction were performed. For soleus and EDL samples, all spectra were normalized to the creatine peak at 3.02 ppm before extraction of raw integral area, to overcome the acquisition instruments difference. These values were utilized for quantitation or as a raw data in Metaboanalyst analysis [41]. To quantitate lipids, 1D NOESY spectra were utilized, while 1D PROJECT spectra were utilized to quantitate the small metabolites (to reduce the possible influence of broad lipid peaks on the peak/s of these small metabolites).

Equations 1 and 2 reported by Mosconi et al. [42]. were used to determine polyunsaturation index (PI) and unsaturation index (UI), using un-normalized peak integral areas. The PI and UI are given as:

1 $$\mathrm{PI}=\:\frac{Lipid\:Y}{\left(\frac{2}{3}\right)Lipid\:X}$$

2 $$\mathrm{UI}=\:\frac{Lipid\:Z}{\left(\frac23\right)Lipid\:X}$$

Here, Lipid X represents the CH_3_ peak at 0.9 ppm, Lipid Y is HC = CH-CH_2_-HC = CH peak at 2.75 ppm, and Lipid Z is the HC = CH peak at 5.4 ppm (Figure S2).

### Assignment of the metabolites

One-dimensional NOESY spectra were used to assign metabolites. Figure S5 shows the assignment of metabolites in whole and extracted serum samples. Correlation spectroscopy (COSY) was collected for a sample using the standard pulse program library of Bruker to verify the metabolites/lipids in solution NMR or in HR-MAS experiments (Supplementary Figure S9). Verification of the metabolites was also done by reviewing literature and biological magnetic resonance bank (BMRB) data [43, 44].

### Data analysis

Multivariate statistical analyses were conducted to analyze the abundance of metabolites among four groups under this study using ^1^H-NMR data (for both aqueous and lipid phase sera samples). Above pre-processed spectra were further binned (0.001 ppm) and aligned locally in several regions prior to multivariate statistical analysis to adjust chemical shifts variability issues, using MestReNova 14.0.1–23559. Integral areas were extracted for some selected metabolites and utilized as input data in the web-based data processing tool MetaboAnalyst 5.0 for analysis. Probability quotient normalization (PQN) and pareto scaling were also applied to these peak areas to minimize any possible bias that might have introduced during sample processing and instrument handling.

### Statistical analysis

Principal component analysis (PCA) and partial least square discriminant analysis (PLS-DA) were performed using MetaboAnalyst 5.0. PLS-DA models were validated using Q^2^ value. Variable Importance in Projection (VIP) plot obtained from PLS-DA analysis was used to summate distinct metabolites with VIP values > 1, driven by high fat diets.

Results are presented as mean ± standard deviation (S.D.). Significance among the groups was determined by two one-way analysis of variance (ANOVA) (GraphPad Prism (version 8.0.0 for Mac, GraphPad Software, San Diego, California USA, www.graphpad.com) with “*p*” = 0.05 and Tukey’s multiple comparisons test was performed among the groups. “*p*” ≤ 0.05 is denoted with “*”, “*p*” ≤ 0.01 is denoted with “**”, “*p*” ≤ 0.001 is denoted with “***” and “p” ≤ 0.0001 is denoted with “****”.

## Supplementary Information

Supplementary Material 1.

## Data Availability

No datasets were generated or analysed during the current study.
