# Supplementary material for: Comparative lipidomic and metabolomic profiling of mdx and severe mdx-apolipoprotein e-null mice
Source: Skelet Muscle. 2024 Dec 23;14:36. doi: 10.1186/s13395-024-00368-w (PMC11664822; doi:10.1186/s13395-024-00368-w)
Supplement: Supplementary file 1 — Supplementary Material 1. [file 13395_2024_368_MOESM1_ESM.docx]

**Comparative metabolomic and lipidomic profiling of mdx and severe mdx-apolipoprotein E-null mice.**

**SUPPLEMENT**

Ram B. Khattri^1,5^, Abhinandan Batra^2,8^, Zoe White^6^, David Hammers^4^, Terence E. Ryan^5,7^, Elisabeth R. Barton^5,7^, Pascal Bernatchez^6*^, Glenn A. Walter^1*^

*^1^Department of Physiology and Functional Genomics, University of Florida, Gainesville, FL, United States*

*^2^Department of Physical Therapy, University of Florida, Gainesville, FL, United States*

*^4^Department of Pharmacology & Therapeutics, University of Florida, Gainesville, FL, USA*

*^5^Department of Applied Physiology and Kinesiology, University of Florida, Gainesville, FL, United States*

*^6^Department of Anesthesiology, Pharmacology & Therapeutics, University of British Columbia, and St Paul’s Hospital, Vancouver, BC, Canada*

*^7^Center of Exercise Science, University of Florida, Gainesville, FL, United States*

*^8^School of Allied Health, University of Louisiana, Monroe, LA, United States*

**Denotes equal contribution*

*
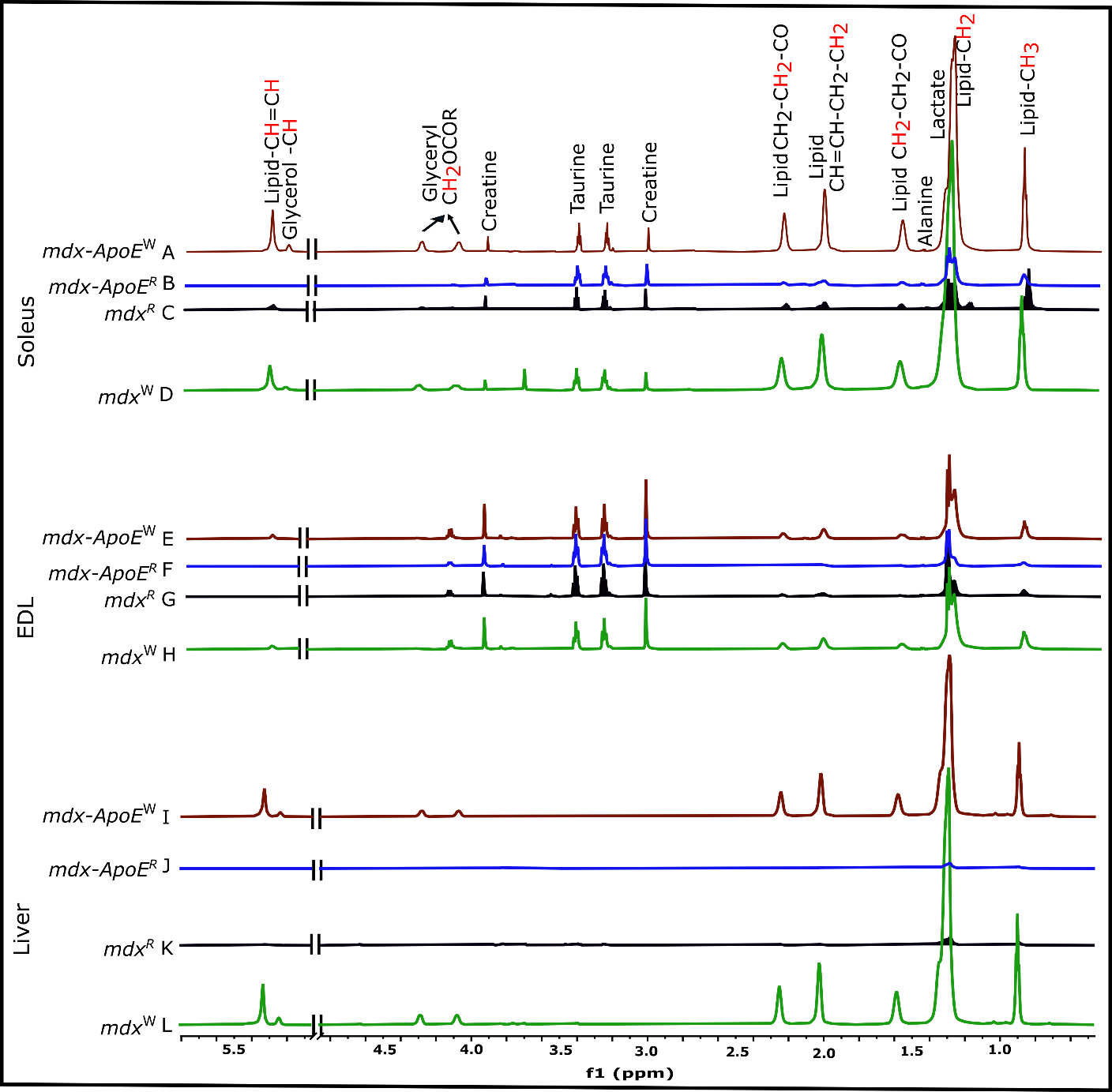
*

**Figure S1.** HR-MAS spectra for soleus, EDL, and liver tissue samples (aliphatic region) showing lipids and small metabolites resonance for four different groups: 1) *mdx-ApoE*^W^ (brown; A, E, & I), 2) *mdx-ApoE*^R^ (blue; B, F, & J), 3) *mdx*^R^ (black; C, G, & K), and 4) *mdx*^W^ (green; D, H, & L). The lipid levels elevated significantly in mice on high fat diet as compared to other regular diet groups. EDL: extensor digitorum longus.


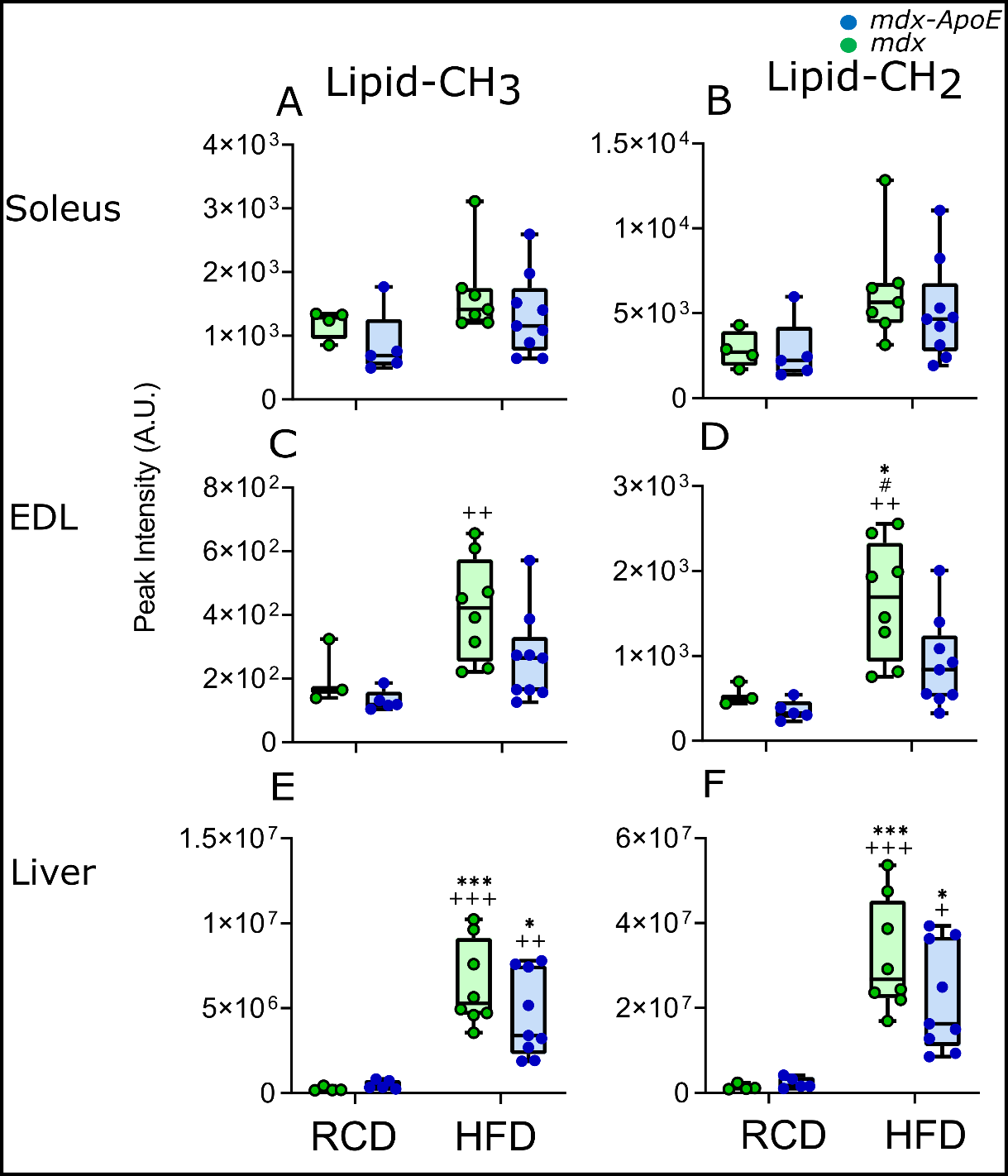


**Figure S2.** Box and Whisker plots showing relative abundance of lipids CH_3_ and lipid CH_2_ for three different tissue samples (soleus, extensor digitorum longus, and liver) via ^1^H HR-MAS spectra. “p” ≤ 0.05 is denoted with “*”, “p” between 0.01-0.001 is denoted with “**”, and “p” ≤ 0.001 is denoted with “***”. “Asterisk/s” means significantly different in regular and high-fat diet within the same strain, “hash tag” means significantly different than the other regular diet strain, and “plus” means significantly different than the other high-fat diet strain. The number of samples per group were as follows: *mdx*-*ApoE*^R^ (n=5), *mdx*-*ApoE*^W^(n=9), *mdx*^R^(n=3-4), and *mdx*^W^(n=7-8). RCD: regular chow diet, HFD: high fat diet, & EDL: extensor digitorum longus.


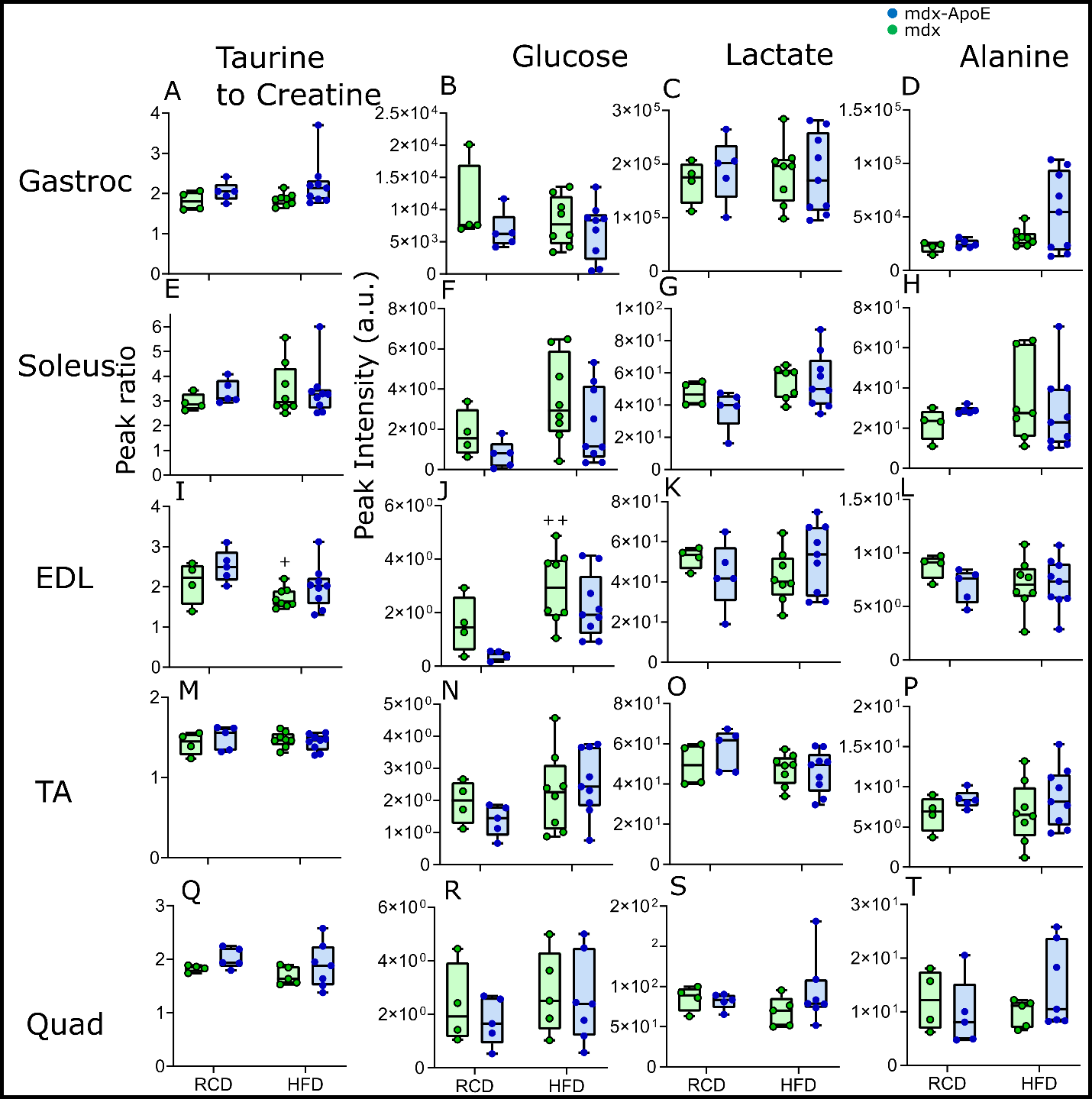


**Figure S3.** Taurine/creatine ratio, glucose, lactate, and alanine level in gastrocnemius, soleus, extensor digitorum longus, tibialis anterior, and quadriceps muscle tissue that change in *mdx* with different diet regimes. Significance was determined by two way ANOVA with “p” = 0.05-0.01, “p” ≤ 0.05 is denoted with “*”/”^#^”/”^+^”, “p” between 0.01-0.001 is denoted with “**”/”^##^”/”^++^”, and “p” ≤ 0.001 is denoted with “***/###”/”^+++^”. “Asterisk/s” means significantly different in regular and high-fat diet within the same strain, “hash tag” means significantly different than the other regular diet strain, and “plus” means significantly different than the other high-fat diet strain. The number of samples per group were as follows: *mdx*-*ApoE*^R^ (n=5), *mdx*-*ApoE*^W^(n=9), *mdx*^R^(n=4), and *mdx*^W^(n=8). RCD: regular chow diet, HFD: high fat diet, gastroc: gastrocnemius, quad: quadriceps, TA: tibialis anterior, and EDL: extensor digitorum longus.

**
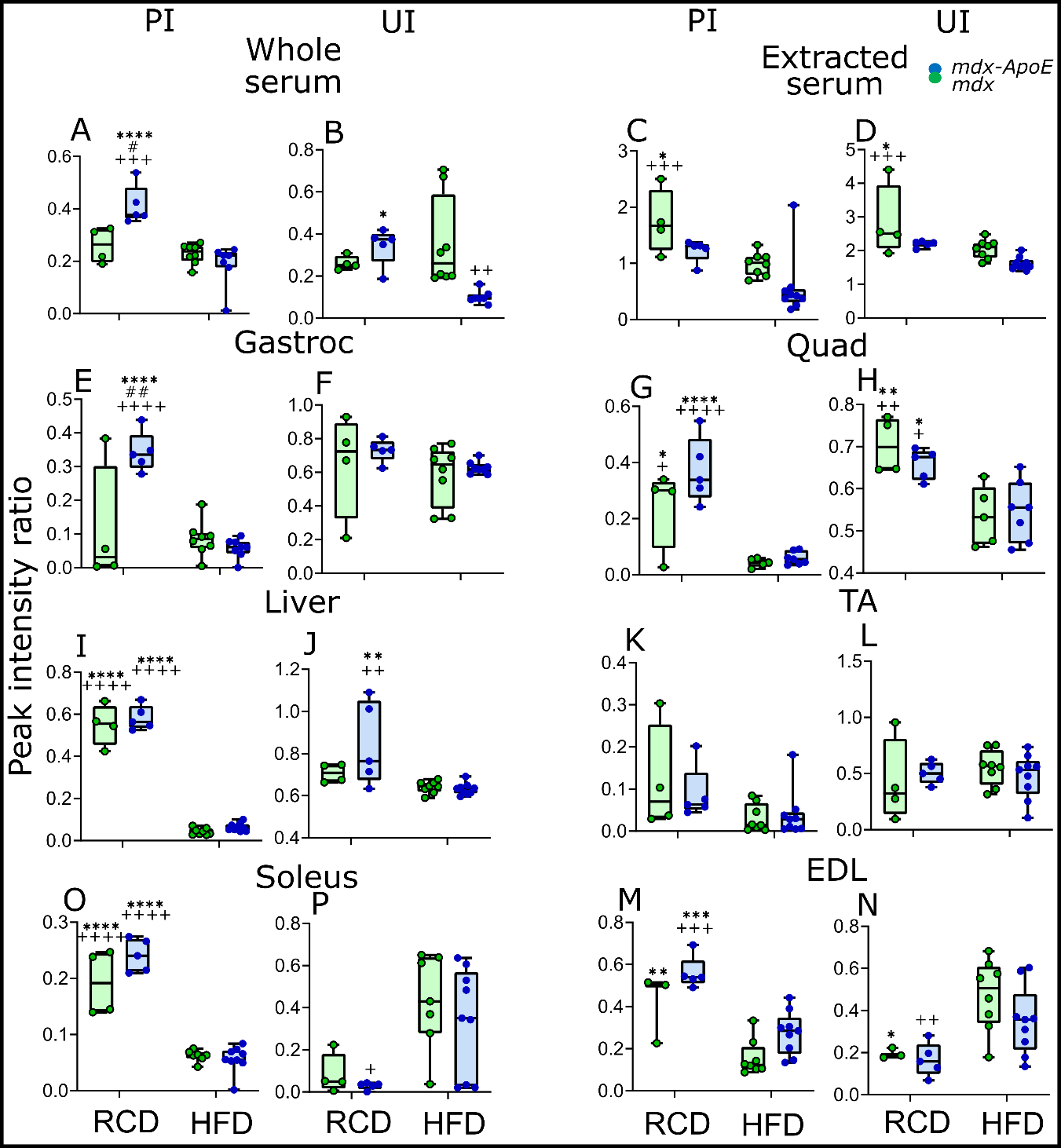
**

**Figure S4:** Poly-unsaturation (PI) and unsaturation (UI) indexes (with error bars) determined for eight different sets of samples via either ^1^H NMR or ^1^H HR-MAS spectra. Significance was determined by two way ANOVA with “p” = 0.05-0.01, “p” ≤ 0.05 is denoted with “*”/”^#^”/”^+^”, “p” between 0.01-0.001 is denoted with “**”/”^##^”/”^++^”, and “p” ≤ 0.001 is denoted with “***/###”/”^+++^”. “Asterisk/s” means significantly different in regular and high-fat diet within the same strain, “hash tag” means significantly different than the other regular diet strain, and “plus” means significantly different than the other high-fat diet strain. The number of samples per group were as follows: *mdx*-*ApoE*^R^ (n=4-5), *mdx*-*ApoE*^W^(n=9), *mdx*^R^(n=4), and *mdx*^W^(n=8).


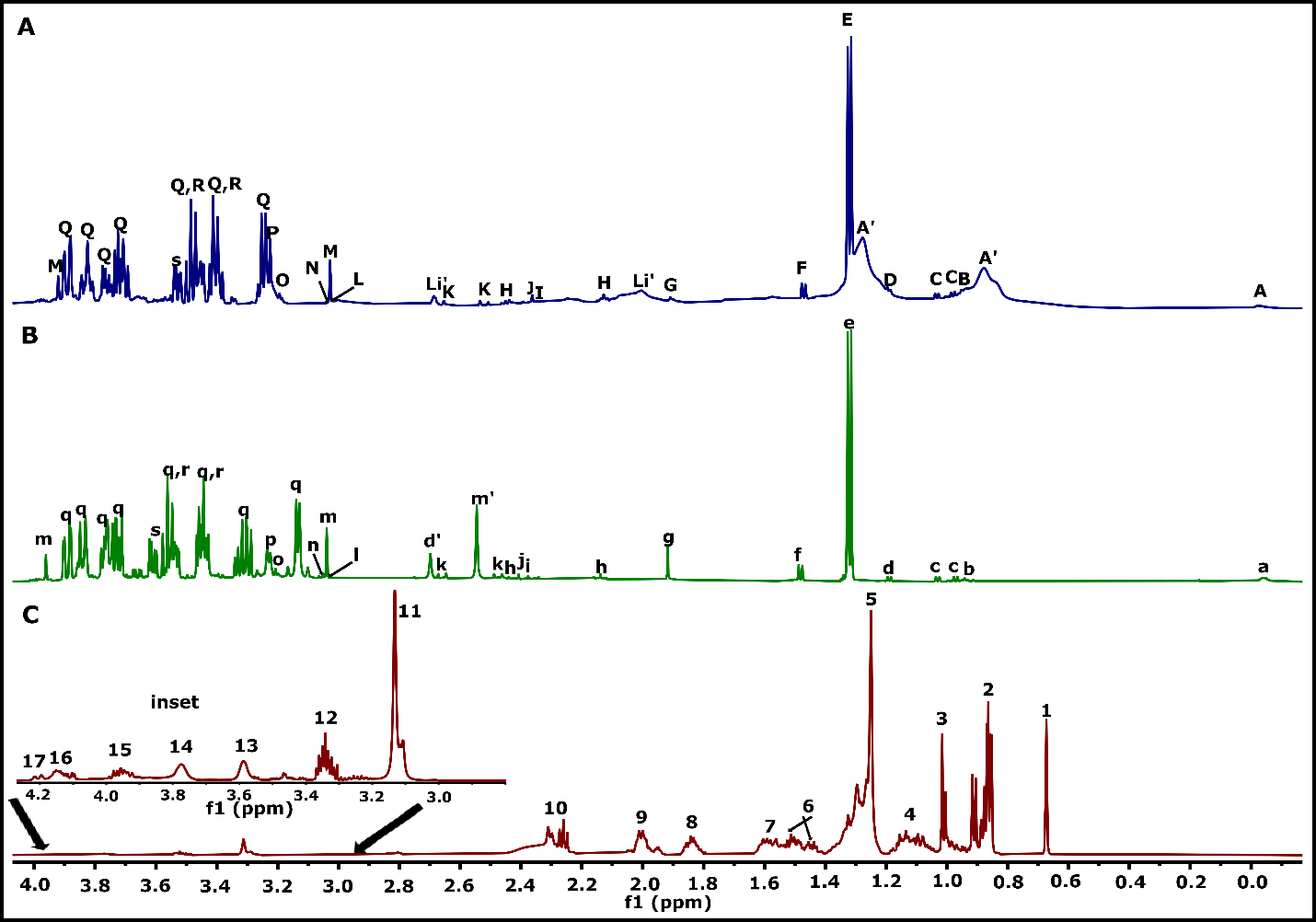


**Figure S5.** NMR serum spectra (aliphatic region) from a mdx mouse model. Un-extracted serum (top) representing the combination of lipoproteins (A’), lipids (Li’), metabolites (B-S), metabolites (middle; a-s) only, lipids (bottom; 1-17) only. A & a represent DSS peak, A’ is lipoprotein, B & b are leucine, C & c are valine, D & d are propylene glycol, E & e are lactate, F & f are alanine, G & g are acetate, H & h are glutamine, I & I are Pyruvate, J & j are Succinate, K & k are citrate, m’ is methylamine (from EDTA), d’ is dimethylamine (from EDTA), L & l are lysine, M & m are unknown, N & n are creatinine, O & o are unknown, P & p are unknown, Q & q are glucose, R & r are taurine, and S & s are glycerol. (C), 1 is (CH_3_) cholesterol/cholesterol ester (C18) , 2 is (CH_3_) cholesterol/esterified and free fatty acids, 3 is (CH_3_) cholesterol (C19), 4 is cholesterol, 5 is (-CH_2_-CH_2_-CH_2_-) fatty acid, 6 is cholesterol, 7 is (CH_2_-CH_2_-COO-) of phospholipid and triglyceride, 8 is (H16α, H1α, H2β)cholesterol/cholesterol ester, 9 is (CH_2_-CH=CH-CH_2_)phospholipid and triglyceride, 10 is (CH_2_-COO-)phospholipid and triglyceride, 11 is N^+^(CH_3_)_3_, 12 is unknown, 13 is (-CH_2_-N) phosphatidylcholine, 14 is (3CH_2_-) phosphatidylcholine, 15 is (-CH_2_-) triglyceride, 16 is (-CH_2_-) triglyceride, 17 is (1CH-) Phospholipid and triglyceride.

**
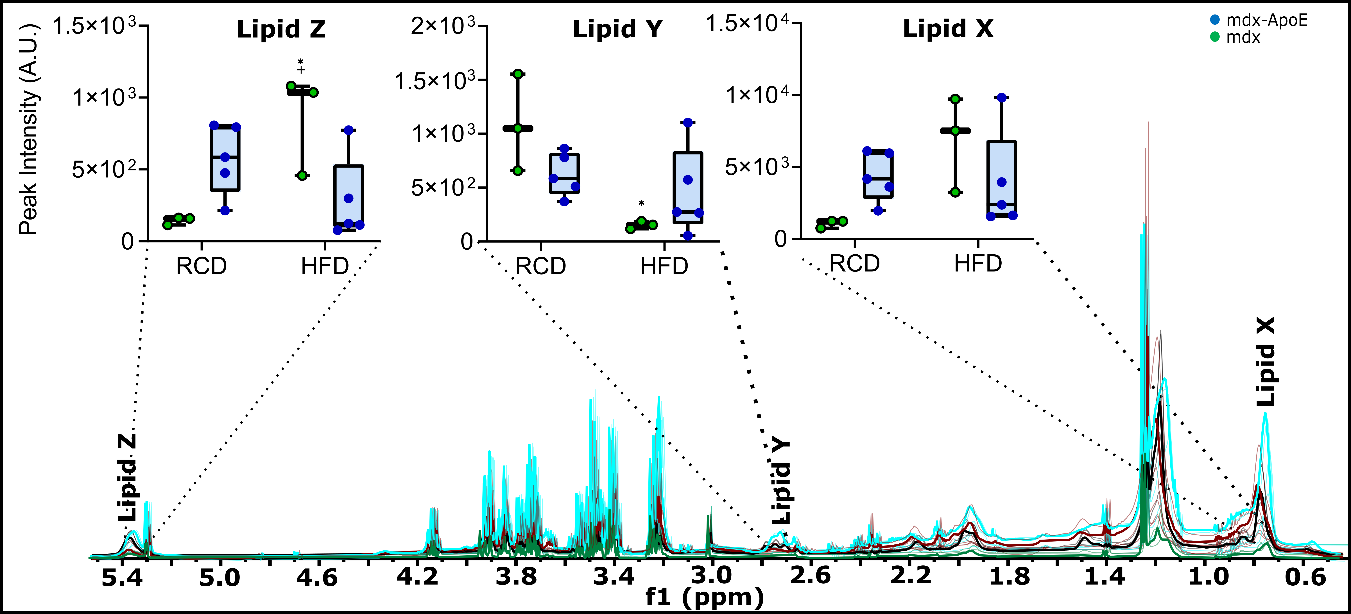
Figure S6.** Whole serum 1D ^1^H spectra showing lipid X or lipid-CH_3_, lipid Y and lipid Z levels in the four groups. Light colors spectra are from individual mice, and the intense colors spectra represent the mean for that group. Significance was determined by two way ANOVA with “p” = 0.05-0.01, “p” ≤ 0.05 is denoted with “*”/”^#^”/”^+^”, “p” between 0.01-0.001 is denoted with “**”/”^##^”/”^++^”, and “p” ≤ 0.001 is denoted with “***/###”/”^+++^”. “Asterisk/s” means significantly different in regular and high-fat diet within the same strain, “hash tag” means significantly different than the other regular diet strain, and “plus” means significantly different than the other high-fat diet strain. The number of samples per group were as follows: *mdx*-*ApoE*^R^ (n=5), *mdx*-*ApoE*^W^(n=5), *mdx*^R^(n=3), and *mdx*^W^(n=4).


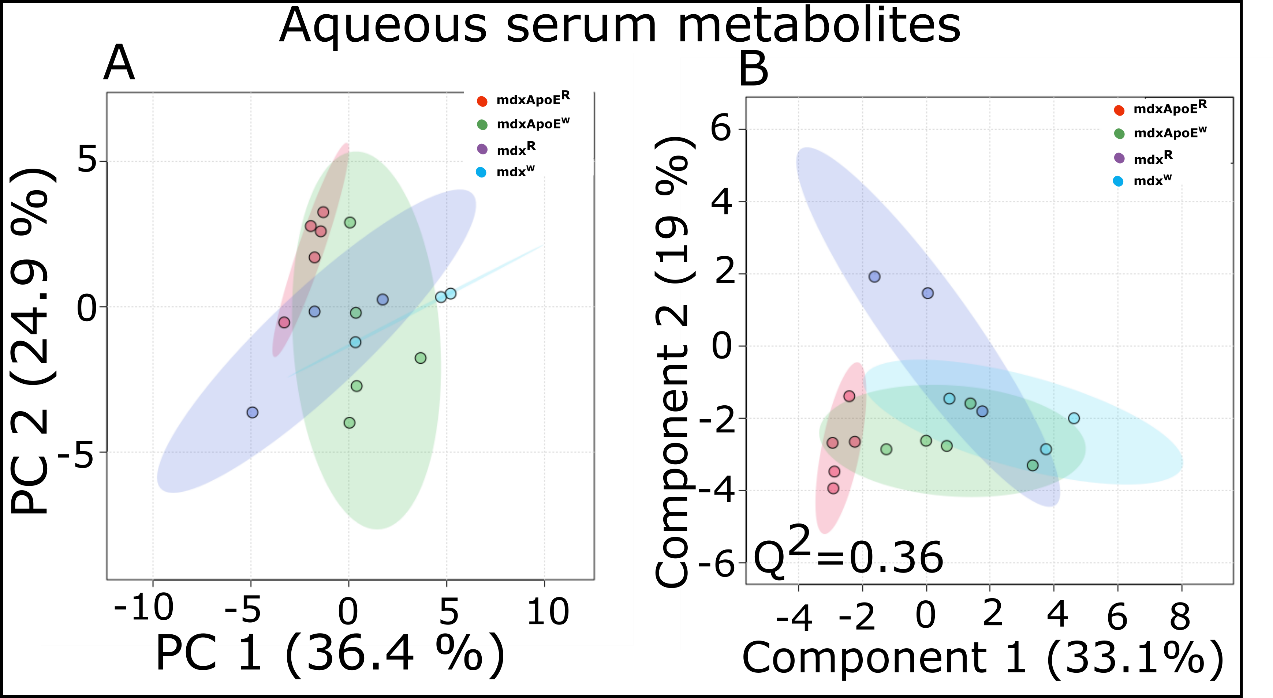


**Figure S7.** ^1^H NMR metabolomics profiles for four diet-dependent groups of mdx mice for aqueous phase sera samples obtained from: A) principal component analysis B) Partial least square discriminant analysis. mdx-apoE^R^ (red), mdx-ApoE^W^(green), mdx^R^ (blue), and mdx^W^ (cyan).

Table ST1. List of chemical shifts/metabolites compelling separation among the four groups obtained from VIP-plot of PLSDA analysis for aqueous phase serum samples, along with their VIP scores value.

| **Spectra range (ppm)** | **Metabolite** | **Peak Pattern** | **VIP Scores** |
| --- | --- | --- | --- |
| **1.30-1.32** | Lactate | S | ~1.68 |
| **8.43-8.45** | Formate | S | ~1.56 |
| **2.68-2.71** | Dimethylamine | S | ~1.54 |
| **0.98-1.00** | Isoleucine | D | ~1.40 |
| **1.89-1.91** | Acetate | S | ~1.38 |
| **1.17-1.20** | Propylene glycol | D | ~1.28 |
| **0.90-0.93** | 2-Aminobutyrate | T | ~1.2 |
| **2.64-2.65** | Methylamine | S | ~1.2 |
| **2.35-2.36** | Pyruvate | S | ~0.98 |
| **5.20-5.23** | Gulcose | D | ~0.86 |
| **1.44-1.47** | Alanine | D | ~0.82 |
| **2.07-2.14** | Glutamine | M | ~0.80 |
| **0.930-0.955** | Leucine | T | ~0.49 |

**
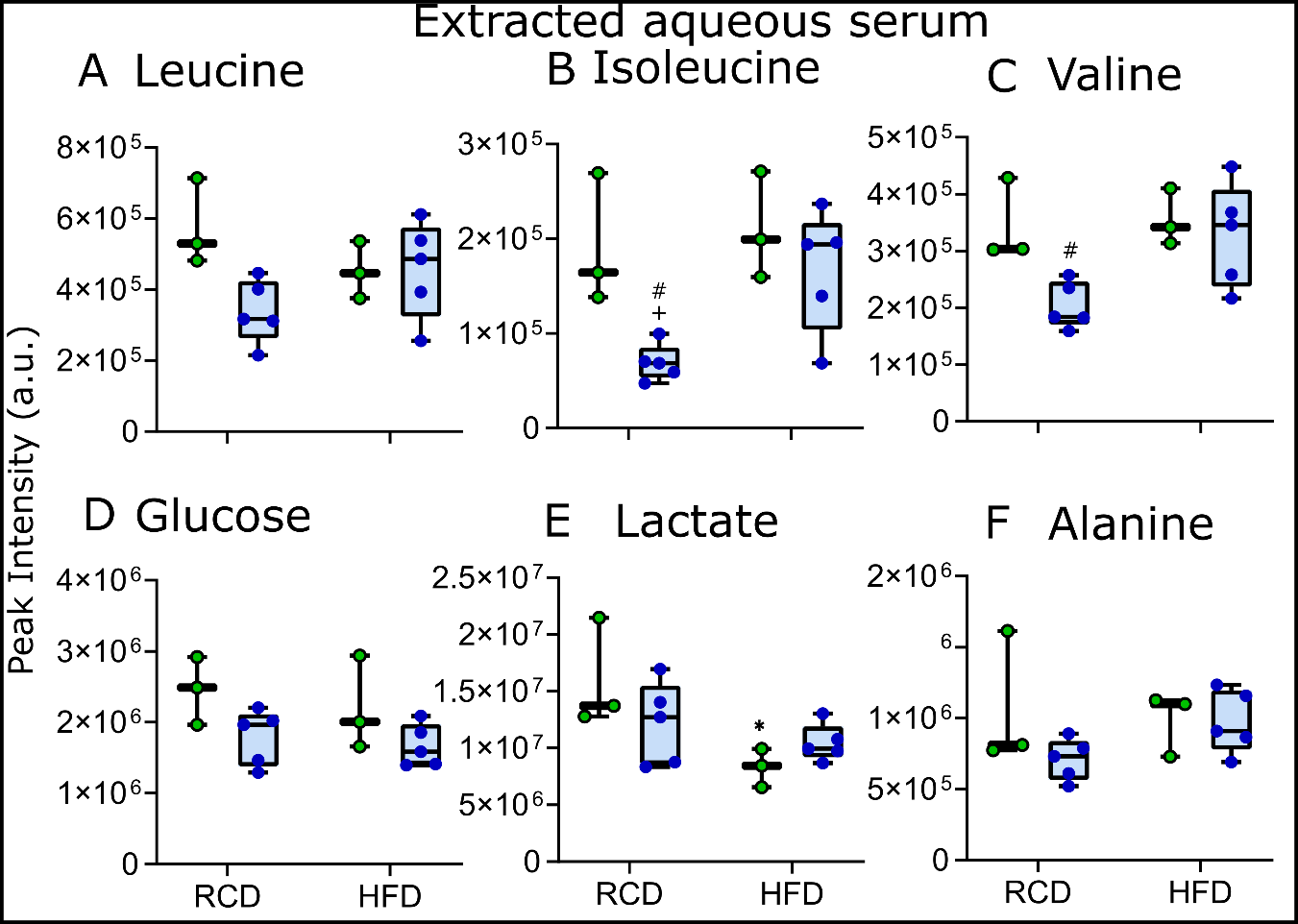
Figure S8.** Branched chain amino acids (leucine, isoleucine, valine), glucose, lactate, and alanine level in extracted aqueous phase serum samples that change in *mdx* with different diet regimes. “p” ≤ 0.05 is denoted with “*”, “p” between 0.01-0.001 is denoted with “**”, and “p” ≤ 0.001 is denoted with “***”. “Asterisk/s” means significantly different in regular and high-fat diet within the same strain, “hash tag” means significantly different than the other regular diet strain, and “plus” means significantly different than the other high-fat diet strain. The number of samples per group were as follows: *mdx*-*ApoE*^R^ (n=5), *mdx*-*ApoE*^W^(n=5), *mdx*^R^(n=3), and *mdx*^W^(n=4). RCD: regular chow diet, HFD: high fat diet,

**
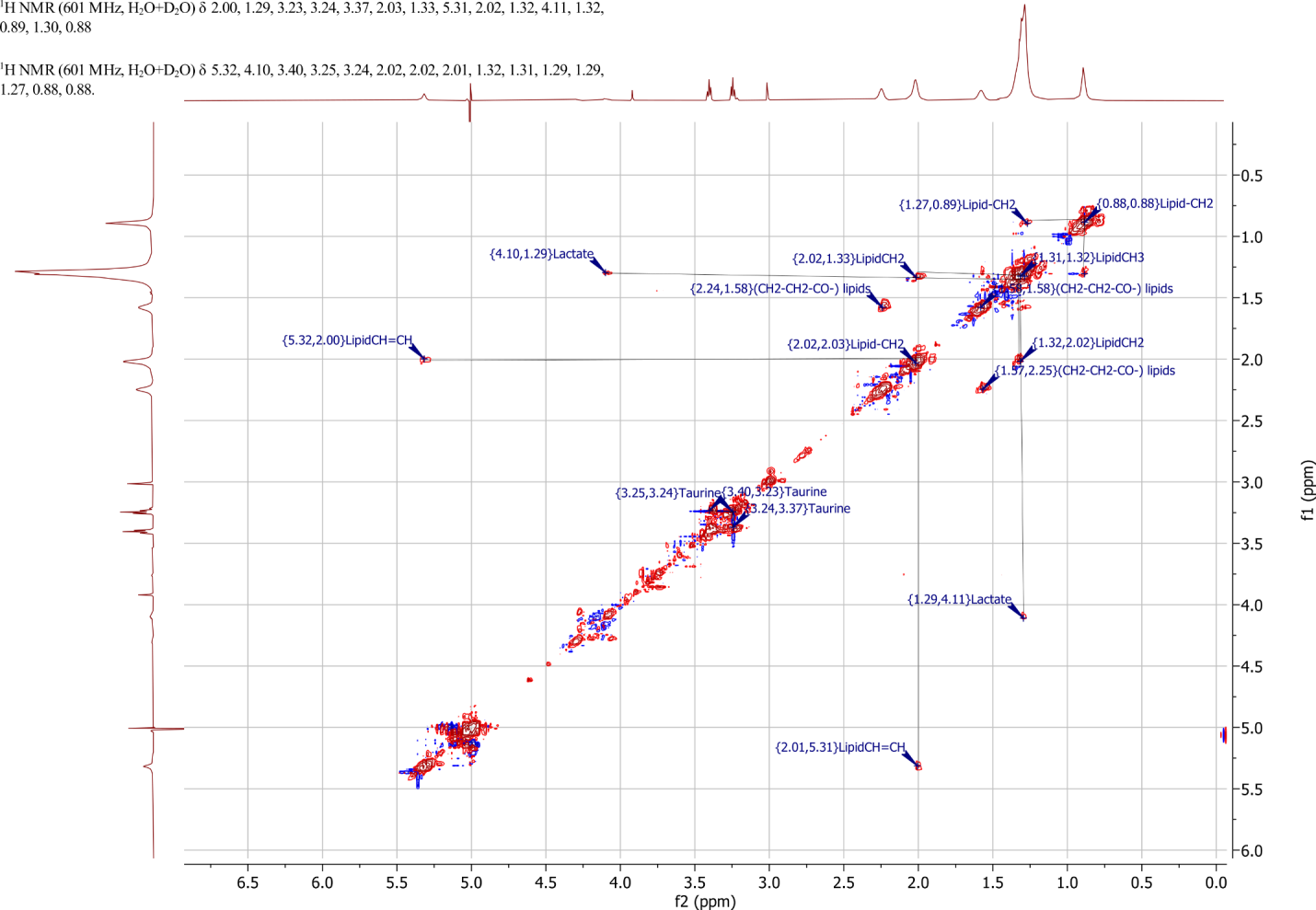
**

**Figure S9.** A portion of COSY spectrum for the muscle sample showing individual metabolites.
